# L‐carnitine increases cell proliferation and amino acid transporter expression via the activation of insulin‐like growth factor I signaling pathway in rat trophoblast cells

**DOI:** 10.1002/fsn3.1607

**Published:** 2020-04-28

**Authors:** Shihai Zhang, Zhihui Wu, Jinghui Heng, Min Tian, Jiaming Chen, Fang Chen, Wutai Guan

**Affiliations:** ^1^ Guangdong Province Key Laboratory of Animal Nutrition Control College of Animal Science South China Agricultural University Guangzhou China; ^2^ College of Animal Science and National Engineering Research Center for Breeding Swine Industry South China Agricultural University Guangzhou China

**Keywords:** IGF‐1 signaling, L‐carnitine, nutrient transporter, reproduction

## Abstract

Early embryo implantation and development is primarily determined by the homeostasis between cellular apoptosis and proliferation as well as placental nutrient transporters. Recent studies showed that L‐carnitine enhances female reproductive performance. However, the potential function of L‐carnitine on placenta is largely unknown. In our study, primary rat trophoblast cells were separated and cultured for 12 hr in medium containing various concentrations of L‐carnitine (0, 1, 10, and 50 mM). Placenta trophoblast cells treated with 50 mM L‐carnitine increased the proportion of cells in S phase of the cell cycle (*p* < .05). In addition, live cell percentage was increased when treated with either 10 mM or 50 mM L‐carnitine, which was accompanied with decreased necrotic cells, late apoptotic cells, and early apoptotic cells (*p* < .05). Compared with the control treatment, the mRNA expression of insulin‐like growth factor I (IGF‐1) and insulin‐like growth factor I receptor (IGF‐1R) was higher in rat placenta trophoblasts treated with either 10 mM or 50 mM L‐carnitine (*p* < .05). Similarly, sodium‐dependent neutral amino acid transporter (SNAT)‐1 and SNAT2 were up‐regulated in both mRNA and protein levels when trophoblast cells were treated with 50 mM L‐carnitine (*p* < .05). Inhibiting downstream targets (Akt or ERK signaling pathways) of IGF‐1 signaling pathway partially blocked the effect the L‐carnitine‐induced increase in protein abundances of SNAT1 and SNAT2. Collectively, our data showed protective role of L‐carnitine on placenta trophoblast cells through the involvement of IGF‐1 signaling pathway.

## Graphical abstract

In this paper, our results showed that L‐carnitine protected placenta trophoblast cells against apoptosis and increased their proliferation. The improvement of amino acid transporters (SNAT1 and SNAT2) in trophoblast cells may be partially induced by the activation of IGF‐1 signaling pathway.

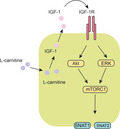

## 1 INTRODUCTION

Dietary nutrient component is a critical factor in the regulation of reproductive performance (Chen et al., 2019; Zeng et al., 2019). The canonical function of L‐carnitine is to regulate the transportation of medium‐chain and long‐chain fatty acids across the inner mitochondrial membrane for β‐oxidation (Sada, Kato, Sumimoto, Ohmori, & Ohdan, 2015). Recent studies have shown that L‐carnitine also participates in the regulation of reproduction. During lactation period, L‐carnitine increases piglets weaning weight through the enhancement of milk production of the sows (Ramanau, Kluge, Spilke, & Eder, 2004b). Sows fed L‐carnitine‐supplemented diets during pregnancy increase the number of piglets born alive and decrease the number of stillborn and mummified pigs (Musser et al., 1999; Ramanau, Kluge, Spilke, & Eder, 2002a, 2004a). Similar results are also observed in models like mice and rat (Agarwal, Sengupta, & Durairajanayagam, 2018). These findings indicate that L‐carnitine might regulate embryo quality and viability, decrease implantation failure and pregnancy loss. However, the underlying mechanism of the beneficial effects of L‐carnitine during this period is still unclear. In this research, we focused on the effects of L‐carnitine on cultured rat trophoblast cells and its underlying mechanism.

Successful implantation and maintenance of placenta during gestation are dependent on balanced cell proliferation and apoptosis (Sharp, Heazell, Crocker, & Mor, 2010). In the placenta, fast proliferation is observed during the early pregnancy and the proliferation diminishes gradually as pregnancy proceeds (Ishihara et al., 2000). Conversely, apoptosis ratio is low after conception and is increased temporarily before the parturition (Smith, Baker, & Symonds, 1997). The imbalance between apoptosis and proliferation might result in various pathological changes, such as spontaneous abortion and intrauterine growth retardation (IUGR; Longtine, Chen, Odibo, Zhong, & Nelson, 2012; Luo et al., 2010; Sun et al., 2013). Regulating the homeostasis between proliferation and apoptosis in the placenta plays an important role in the fetus development. Therefore, L‐carnitine might be a key element to maintain the homeostasis between apoptosis and proliferation in trophoblast cells.

Placental nutrient transportation is also of fundamental importance for fetal growth, as fetal development is largely dependent on nutrients from maternal circulation. Amino acids have various potential functions in fetoplacental development (Regnault, Friedman, Wilkening, Anthony, & Hay, 2005). Placenta trophoblast cell is a useful model to study placental function, for example, nutrient transportation (Hu & Cross, 2011). In the placenta, neutral amino acids are mainly transported by the system A sodium‐dependent neutral amino acid transporter (SNAT), which consists of three isoforms (SNAT 1, SNAT 2 and SNAT 4; Desforges et al., 2009; Glazier & Sibley, 2006; Jones, Powell, & Jansson, 2007), whereas cationic amino acids (lysine, arginine, and histidine) are mainly transported by the cationic amino acid transporter‐1 (CAT‐1), which is a major cationic transport system in the placenta (Ayuk, Sibley, Donnai, D'Souza, & Glazier, 2000). Glucose is the principal carbohydrate transported from maternal circulation to fetus (Langdown & Sugden, 2001). In the mammalian placenta, GLUT1 and GLUT3 are the two major glucose transporter isoforms (Lager & Powell, 2012). GLUT1 is predominantly expressed in the placenta during early pregnancy, which is gradually replaced by GLUT3 during late gestation (Baumann, Deborde, & Illsley, 2002; Janzen et al., 2013). A key question is whether L‐carnitine could modulate fetal growth through regulating placental glucose and amino acid transporter expression.

Activation of insulin‐like growth factor 1 (IGF‐1) signaling pathway is a possible mechanism of how L‐carnitine regulates reproduction. Previously, L‐carmine was reported to increase maternal IGF‐1 concentration and induce the development of fetus (Eder, 2009). In addition, IGF‐1 increases blastocyst formation and have anti‐apoptotic effect during early embryo development (Lin, Yen, Gong, Hsu, & Chen, 2003; Mandl, Haas, Bischof, Nöhammer, & Desoye, 2002). Although liver is the major organ for IGF‐1 secretion, placenta is also considered as one of dominant sources of IGF‐1 during gestation (Martín‐Estal, De La Garza, & Castilla‐Cortazar, 2016). Furthermore, trophoblast cells were also found to produce IGF‐1 locally (Adams, 2002). Therefore, the primary objective of this study was to evaluate the effects of L‐carnitine on the proliferation and apoptosis, nutrient transporter systems and activation of IGF‐1 signaling pathway in rat placental trophoblast cells.

## 2 MATERIALS AND METHODS

### 2.1 Isolation and purification of placenta trophoblast cell

Placenta tissues were collected under sterile conditions from gestational day 11 *SD* rats and were cut into small cubic masses (1 mm^3^). Dissected tissues were digested with 0.25% trypsin (Gibco) and 0.2% collagenase (Sigma Chemical Co.) for 5 min at 37°C, and subsequently treated with 200 U/ml DNase I (Sigma Chemical Co.) for 5 min at 37°C. Any undigested fragments were removed and the cells were then collected by centrifugation (300 *g*) and washed with DMEM. To further purify the cells, trophoblasts were isolated by Percoll gradient centrifugation. The Percoll gradients were made from 45% to 15% Percoll (V/V) in every 5% steps of 5.0 ml each. Trophoblasts were collected in the density of the gradient between 1.048 and 1.062 g/ml. To eliminate the contamination of red blood cells, 5.0 ml of lysis buffer (155 mM NH_4_Cl, 10 mM KHCO_3_, and 0.14 mM EDTA (pH 7.2)) was added to the cell pellets. Isolated cells were verified by using immunohistochemical staining (stained for cytokeratin and vimentin; Figure S1).

### 2.2 Cell culture and treatment

Verified trophoblast cells were seeded in six‐well plates. After reaching 90% confluency, cells were starved for 4 hr in a fetal calf serum (FCS)‐free DMEM medium (GIBCO, Thermo Fisher Scientific). After starvation, cells were cultured in DMEM medium containing 5% FCS with 0, 1, 10, or 50 mM L‐carnitine for 12 hr. For experiment using inhibitors, trophoblast cells were pretreated with 10 μM PD098059 (Sigma) or LY294002 (Sigma) for 30 min.

### 2.3 Flow cytometric analysis of cell cycle and cell apoptosis

After treated with L‐carnitine, cells were washed twice with PBS and then stained according to Annexin V‐FITC/PI kit protocol (BD PharMingen). Briefly, cells were incubated with Annexin V‐FITC and propidium iodide (PI; Sigma‐Aldrich) and incubated for 15 min in the dark. Samples were read on a FACScan flow cytometer (Becton Dickinson).

After treated with L‐carnitine, cells were treated with 0.25% trypsin (Sigma‐Aldrich) and detached from the plate. Cells were stained with propidium oxide (BD Biosciences). The distribution of the cells in G0/G1, S, and G2/M phases were determined with FACScan cytometry (Becton Dickinson).

### 2.4 RNA isolation and RT‑PCR analysis

Total RNA was isolated using TRIzol (Life Technologies) according to the manufacturer's instruction. Total RNA was reverse‐transcribed to complementary DNA (cDNA) using a PrimeScript 1st Strand cDNA Synthesis Kit (Takara) according to the manufacturer's protocol. Primers for the selected genes were designed using Primer Premier 5.0. Real‐time PCR was performed using an ABI PRISM^®^7500 with SanTaq Plus PCR Master Mix (Sangon, Biotech) containing 3 mM MgCl_2_, 0.2 mM dNTP, and 0.2 U/µl Pfu DNA polymerase. The PCR system consisted of 10.0 μl of SanTaq Plus PCR Master Mix, 5.0 μl of cDNA (1:20), 4 μl of double distilled water, and 1 μl of primer pairs (25 μM forward and 25 μM reverse) in a total volume of 20 μl. The protocols for all genes included a denaturation program (3 min at 95°C), amplification and quantification program repeated for 35 cycles (15 s at 95°C, 15 s at 60°C, 40 s at 72°C), followed by the melting curve program at 60–95°C with a heating rate of 0.1°C per second and continuous fluorescence measurement. Quantification of target mRNA was determined by the delta–delta CT method (^2−ΔΔ^CT), using GAPDH as the housekeeper to normalize each sample. The primers are listed in Table S1.

### 2.5 Western blot

All samples were homogenized in RIPA Lysis Buffer (Beyotime). Protein concentration was measured using a BCA Protein Assay Kit (Beyotime). About 30 μg of protein from each sample was separated on SDS‐PAGE gels and then transferred to nitrocellulose membranes. After incubation with 5% skimmed milk for 1 hr at room temperature, membranes were incubated with primary antibody overnight at 4°C. The antibodies used in this experiment were listed as follows: SNAT1 (1:1,000, Santa Cruz Biotechnology), SNAT2 (1:1,000, Santa Cruz Biotechnology), p‐mTOR (1:1,000, Cell Signaling Technology), mTOR (1:1,000, Cell Signaling Technology, USA), p‐ERK (1:1,000, Cell Signaling Technology), ERK (1:1,000, Cell Signaling Technology), p‐Akt (1:1,000, Cell Signaling Technology), Akt (1:1,000, Cell Signaling Technology), and Actin (1:1,000, Cell Signaling Technology).

### 2.6 Statistical analysis

Statistical analyses were performed using the statistical software SAS version 9.2. Data were analyzed by ANOVA according to the GLM procedure of SAS. Differences among treatment were determined using Duncan's multiple range test. *p* value less than .05 was considered significant.

## 3 RESULTS

### 3.1 Effects of L‐carnitine on the cell cycle in rat placenta trophoblasts

**Figure 1:**
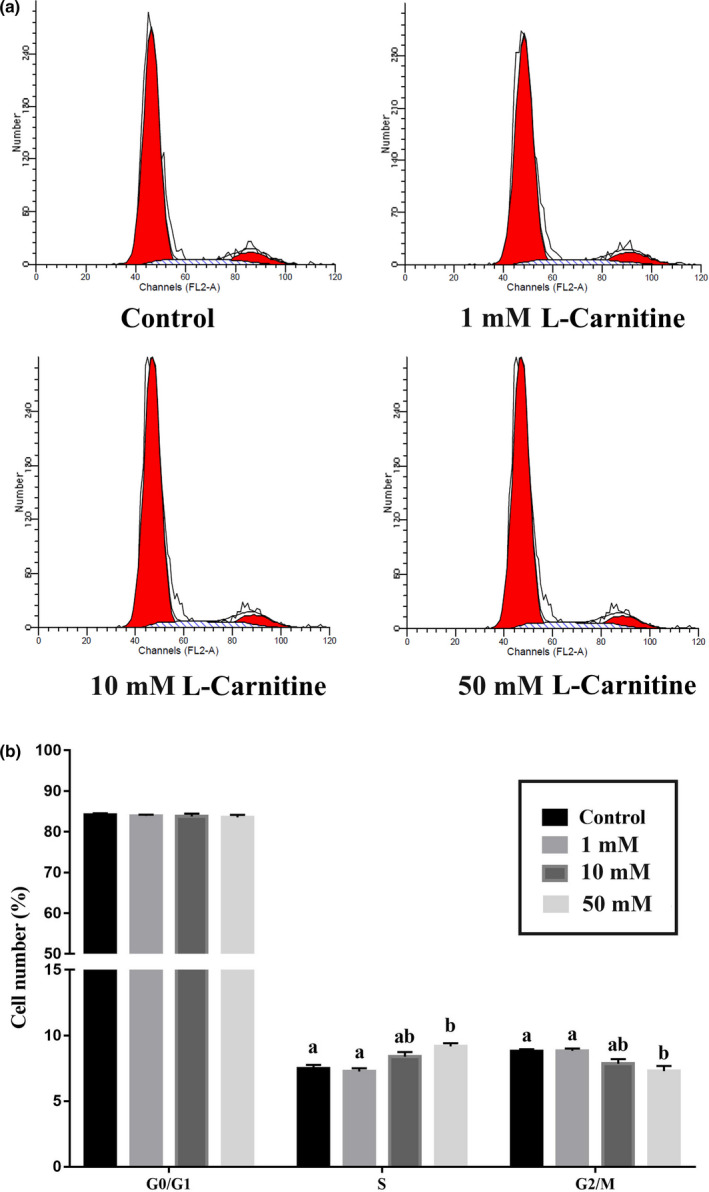
Effect of L‐carnitine on cell cycle distribution in rat placenta trophoblast cells. (a) Flow cytometric analysis of rat placenta trophoblast cells treated with 0, 1, 10, or 50 mM L‐carnitine. (b) Data from the cell cycle distribution were summarized and presented as the means ± SE from three independent experiments. Bars with different letters are significant different (*p* < .05)

The effect of L‐carnitine on the cell cycle of the rat placenta trophoblasts was determined by flow cytometry. As shown in Figure 1, more cells were in the S phase of cell cycle when treated with 50 mM L‐carnitine for 12 hr, with a concomitant decrease in the proportion of cells in the G2/M phase (*p* < .05).

### 3.2 Effects of L‐carnitine on apoptosis in rat placenta trophoblasts

**Figure 2:**
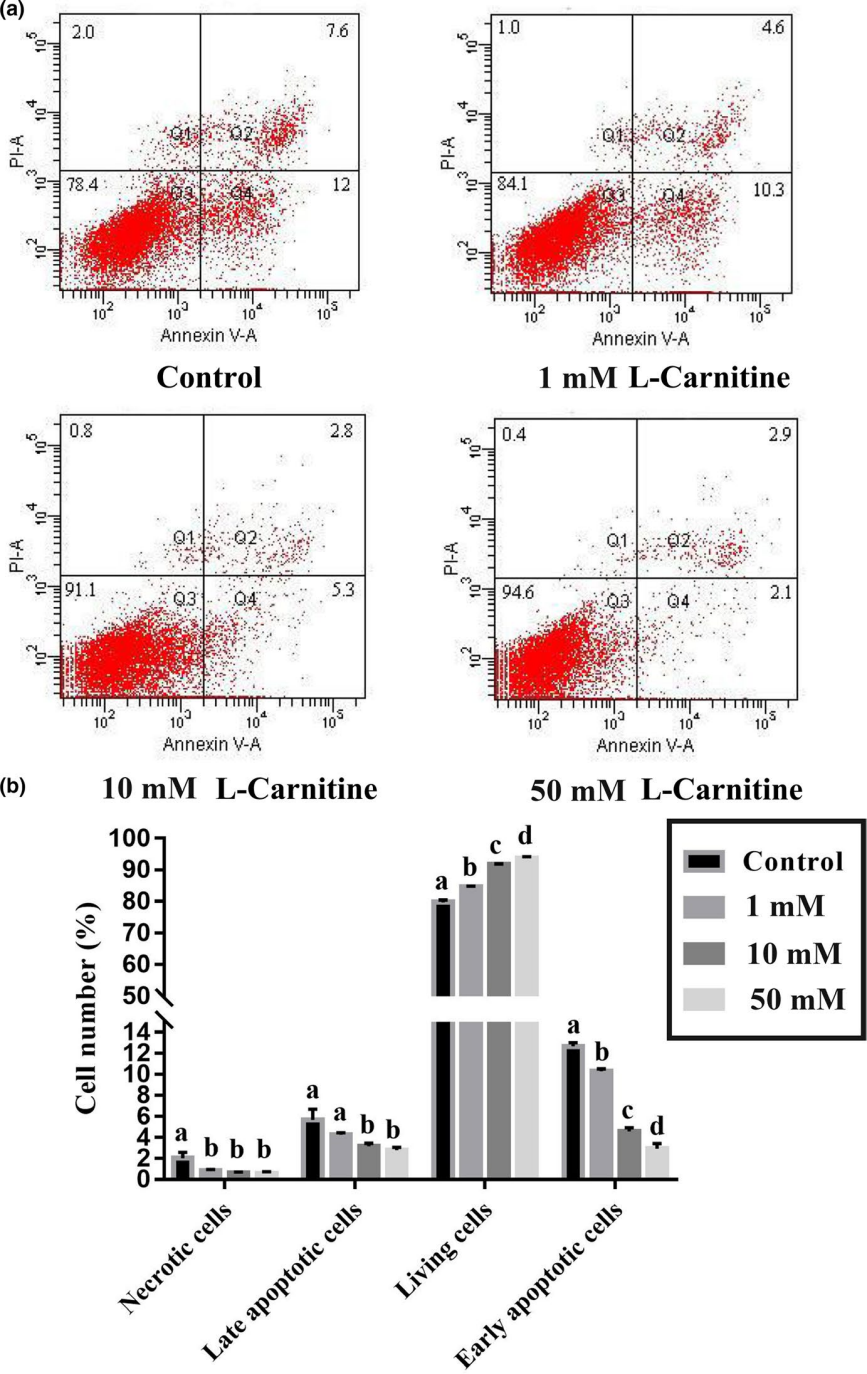
Scatter plots of Annexin V‐FITC/PI staining in control or cells treated with various concentrations of L‐carnitine (0, 1, 10 or 50 mM) in a quadrant analysis. Note: Q1‐necrotic or dead cells (Annexin‐FITC−/PI+), Q2‐late apoptotic or dead cells (Annexin‐FITC+/PI+), Q3‐live cells (Annexin‐FITC−/PI−), Q4‐early apoptotic cells (Annexin‐FITC+/PI−). Bars with different letters are significant different (*p* < .05)

The effect of L‐carnitine on apoptosis of placental trophoblasts is presented in Figure 2. The percentage of necrotic cells, late apoptotic cells, and early apoptotic cells were significantly lower in trophoblasts treated with either 10 mM or 50 mM L‐carnitine (*p* < .05). Furthermore, the percentage of live cells was higher when cells were treated with L‐carnitine (*p* < .05).

### 3.3 Effects of L‐carnitine on gene expression in rat placenta trophoblasts

**Figure 3:**
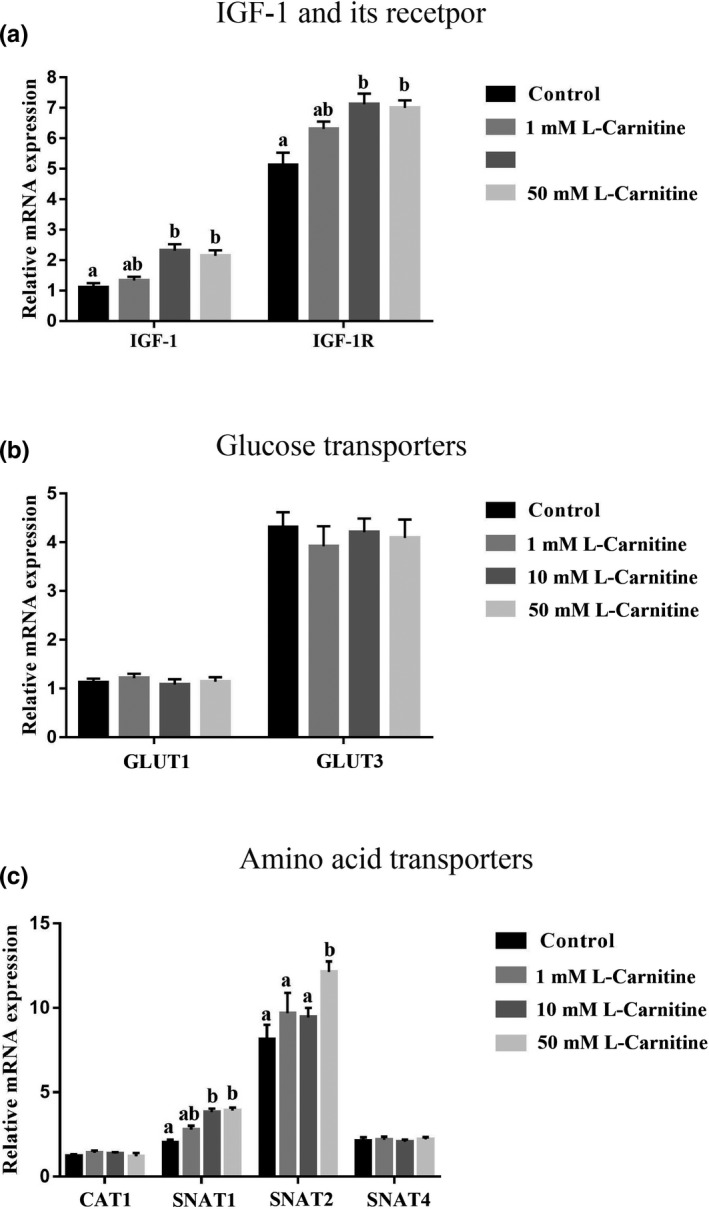
Effects of various concentrations of L‐carnitine (0, 1, 10, or 50 mM) on mRNA expression of IGF‐1 and IGF‐1R (a), glucose transporters (b) and amino acid transporters (c) in rat placenta trophoblast cells. Bars with different letters are significant different (*p* < .05)

Compared with control group, the mRNA expression of IGF‐1 and IGF‐1R was higher in rat placenta trophoblast cells treated with either 10 mM or 50 mM L‐carnitine (*p* < .05; Figure 3a). L‐carnitine did not affect GLUT1 or GLUT3 mRNA expression (Figure 3b). SNAT1 mRNA expression was up‐regulated in cells treated with either 10 or 50 mM L‐carnitine, whereas SNAT2 was only increased when cells were treated with 50 mM L‐carnitine (Figure 3c).

### 3.4 Effects of L‐carnitine on protein abundances in rat placenta trophoblasts

**Figure 4:**
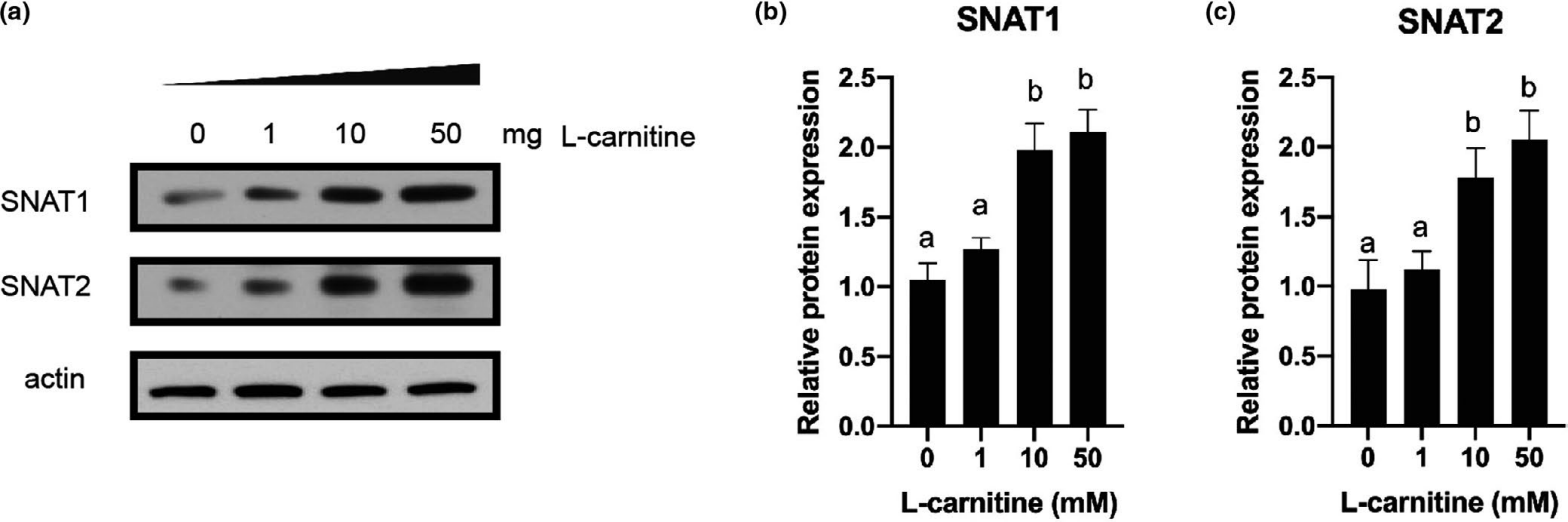
Effect of various concentrations of L‐carnitine (0, 1, 10, or 50 mM) on protein abundances of SNAT1 and SNAT2 in rat placenta trophoblast cells. Bars with different letters are significant different (*p* < .05)

**Figure 5:**
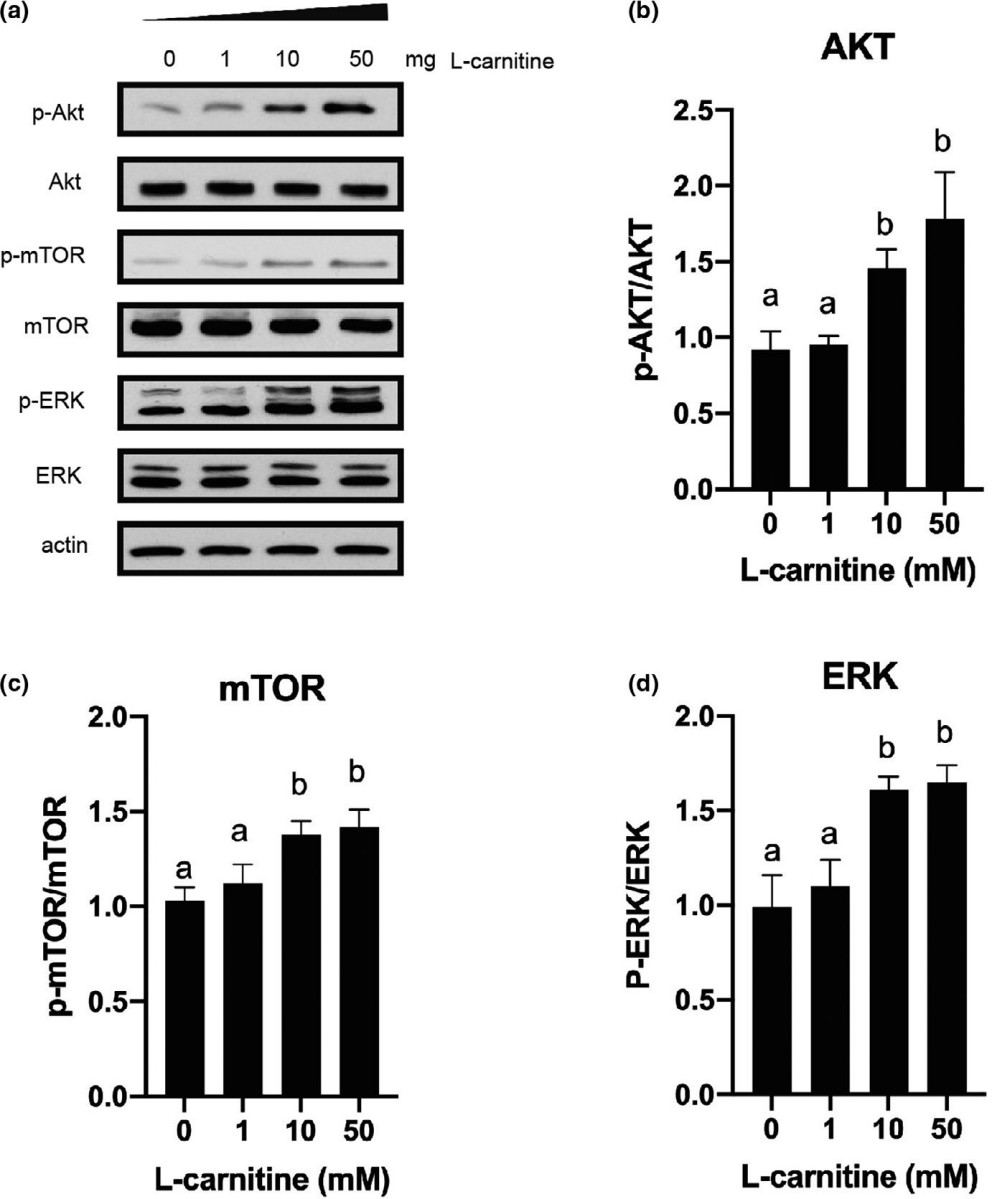
Effect of various concentration of L‐carnitine (0, 1, 10 or 50 mM) on PI3K/Akt/mTOR and ERK signaling pathways in rat placenta trophoblast cells. Bars with different letters are significant different (*p* < .05)

Consistent with the mRNA results, the protein abundances of SNAT1 and SNAT2 were up‐regulated in cells treated with either 10 mM or 50 mM L‐carnitine (*p* < .05; Figure 4). As the mRNA expression of IGF1 and IGF1‐R is increased when cells were treated with L‐carnitine, the IGF‐1 related signaling pathways were detected in our experiments. Trophoblasts treated with either 10 mM or 50 mM L‐carnitine increased phosphorylation of Akt, mTOR and ERK1/2 (*p* < .05; Figure 5).

### 3.5 Effects of L‐carnitine on protein abundances in rat placenta trophoblasts pretreated with IGF‐1 signaling pathway inhibitors

**Figure 6:**
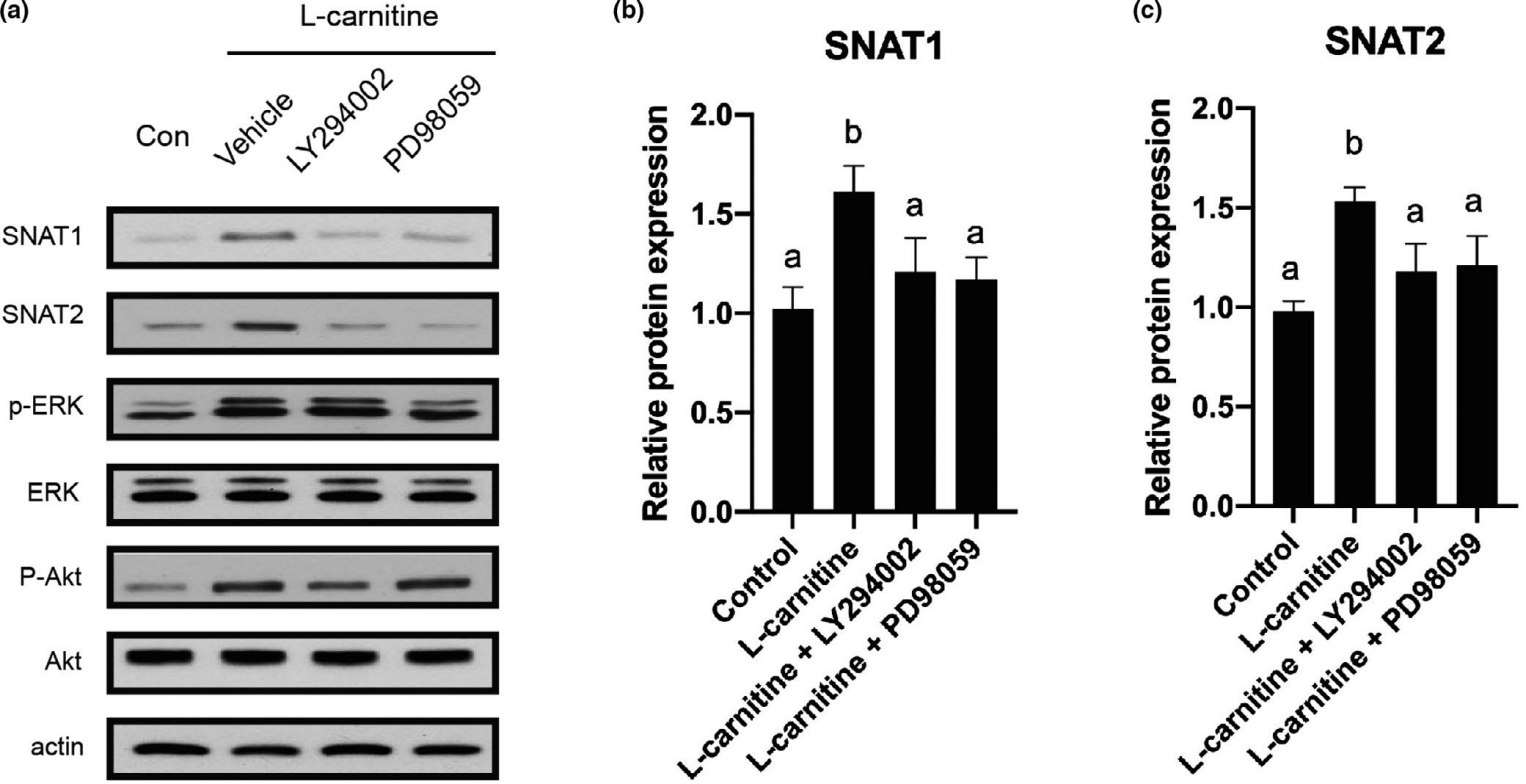
Effect of L‐carnitine on protein abundances of SNAT1 and SNAT2 in rat placenta trophoblast cells pretreated with ERK or PI3K inhibitors. Bars with different letters are significant different (*p* < .05)

PI3K and ERK are two critical downstream signaling pathways of IGF‐1 signaling. PD098059 and LY294002 are the inhibitors of ERK and PI3K, respectively. Cells pretreated with PD098059 or LY294002 both partially rescued the effect of L‐carnitine on the protein abundances of SNAT1 and SNAT2 (*p* < .05, Figure 6).

## 4 DISCUSSION

The anti‐apoptotic function of L‐carnitine has been widely studied in different cell lines. In mouse osteoblastic cells (MC3T3‐E1 cells), L‐carnitine protects cells from apoptosis by inhibiting cytochrome c release and caspase‐3 and caspase‐9 activation (Xie et al., 2008). In neuronal cells, L‐carnitine has similar anti‐apoptotic actions and thus has neuroprotective effect (Ishii, Shimpo, Matsuoka, & Kinoshita, 2000). In skeletal muscle cells, L‐carnitine prevents apoptosis and protects muscle from metabolic injuries (Vescovo et al., 2002). Similar anti‐apoptotic function of L‐carnitine is also observed in lymphocytes (Moretti et al., 2002; Wang et al., 2007). In the present study, we showed that L‐carnitine protected rat placenta trophoblast cells against serum deprivation‐induced apoptosis. Dietary supplementation of L‐carnitine has been shown to increase the birth weight of piglets at weaning (Ramanau, Kluge, Spilke, & Eder, 2002b) and decrease the number of stillborn and mummified pigs (Musser et al., 1999). The trophoblasts from placentas of pregnancies with IUGR exhibit increased apoptosis compared with those from control pregnancies (Levy et al., 2002). Excessive injury of the villous trophoblast layer caused by apoptosis reduces the functional mass of syncytiotrophoblast in IUGR and limits the capacity of nutrient transportation in the villi (Scifres & Nelson, 2009). Therefore, L‐carnitine supplementation during pregnancy might decrease the number of IUGR piglets by protecting placenta trophoblast cells against apoptosis.

The proliferation of trophoblast cells has been considered to be involved in embryo implantation and trophoblast invasion, which is one of the limiting factors for the establishment of pregnancy (Magariños, Sánchez‐Margalet, Kotler, Calvo, & Varone, 2007). The molecular mechanisms underlying the effect of L‐carnitine on placenta trophoblast cell proliferation remain unknown. Our results showed that L‐carnitine supplementation increased the percentage of cells in S phase, which was accompanied with the decrease of cells in G2/M phase. These results indicate that L‐carnitine can increase cell proliferation, which is consistent with previous findings by Colucci et al. (2005) and Santoro et al. (2005) in osteoblasts and ovary cells.

Besides alterations in apoptosis and proliferation, intrauterine growth restriction is associated with a variety of changes in amino acid and glucose contents from maternal circulation (Jansson, 2005). Therefore, glucose and amino acid transporters were measured in the current study. GLUT1 and GLUT3 are two primary transporters mediating facilitated glucose transfer across the placental barrier in early pregnancy (Jansson, Ylvén, Wennergren, & Powell, 2002). However, in the current study, neither GLUT1 nor GLUT3 expression was changed in trophoblast cells treated with L‐carnitine. Similarly, previous study also found that fetal hypoglycemia in IUGR might not due to changes in placental glucose transporters as GLUTs protein abundances were unaltered (Jansson, Wennergren, & Illsley, 1993). Unlike glucose transporters, changes in amino acid transporters were observed when trophoblast cells were treated with L‐carnitine. Transportation of amino acids from maternal to the fetus is an important process resulting in the increase of amino acid concentrations in the fetal circulation. In IUGR, placental amino acid uptake is largely decreased in both human and experimental animal models (Regnault et al., 2005). In the placenta, neutral amino acids are mainly transported by SNAT1, SNAT2, and SNAT4 (Desforges et al., 2009; Glazier & Sibley, 2006; Jones et al., 2007). In our study, trophoblast cells treated with L‐carnitine significant increased SNAT1 and SNAT2. Thus, the up‐regulation of amino acid transporters might partially help to increase the reproductive function when sows are treated with L‐carnitine‐supplemented diets.

Liver‐derived IGF‐1 is the principal source of IGF‐1 in the blood (Sjögren et al., 1999). IGF‐1 exerts its function in both endocrine and autocrine/paracrine manners (Adams, 2002). Trophoblasts not only express IGF‐1 receptor, but also produce IGF‐1 locally. Thus, the effective concentration of this hormone is probably greater in the placenta (Han, Bassett, Walton, & Challis, 1996). Until now, it is still not clear about the physiological role of IGF‐1 during pregnancy, which could be a key player in the regulation of the embryo implantation as well as its maintenance. In this research, we demonstrated that the expression of IGF‐1 and IGF1‐R was up‐regulated in trophoblast cells treated with L‐carnitine. Activation of IGF‐1 signaling pathway stimulates phosphoinositide‐3 kinase (PI3‐K)/Akt and Ras/Raf/MEK/ERK pathways (Denduluri et al., 2015; Hakuno & Takahashi, 2018; Higashi, Sukhanov, Anwar, Shai, & Delafontaine, 2012). It has been well established that phosphorylation of Akt triggers the activation of mTORC1 signaling pathway. Interestingly, recent study also found that ERK phosphates and inhibits tuberous sclerosis (TSC), and further activates mTOR signaling pathway (Ma, Chen, Erdjument‐Bromage, Tempst, & Pandolfi, 2005; Mendoza, Er, & Blenis, 2011), which indicates a potential cross talk between these two pathways. mTOR is a master regulator of a number of biological processes such as protein synthesis (via S6K1; Wang & Proud, 2006; Zhang et al., 2014), translation (via 4EBP1; Fingar et al., 2004; Proud, 2004) and autophagy (via ULK1; Jung, Ro, Cao, Otto, & Kim, 2010; Kim, Kundu, Viollet, & Guan, 2011). Thus, L‐carnitine might regulate SNAT1 and SNAT2 expression through the activation of mTOR signaling pathway. As expected, both Akt and ERK signaling pathways were activated in trophoblast cells supplemented with L‐carnitine. In addition, inhibition of Akt or ERK signaling pathway partially blocked the protein abundances of SNAT1 and SNAT2. Coincidently, IGF‐1 has been reported to stimulate amino acid uptake in placental trophoblasts (Karl, 1995). Our results indicate that L‐carnitine‐induced up‐regulation of SNAT1 and SNAT2 might be partially due to the activation of IGF‐1 signaling pathway. Future studies are needed to clarify other potential signaling pathways that could participate in L‐carnitine regulation.

## 5 CONCLUSIONS

Collectively, our results showed that L‐carnitine protected placenta trophoblast cells against apoptosis and increased their proliferation. The improvement of amino acid transporters (SNAT1 and SNAT2) in trophoblast cells may be partially induced by the activation of IGF‐1 signaling pathway.

## CONFLICT OF INTEREST

All authors declare that they have no competing interest in the present work.

## AUTHORS’ CONTRIBUTIONS

SH Zhang and WT Guan designed the experiment and supervised the project. ZH Wu, JH Heng, M Tian and JM Chen, F Chen performed the experiments and conducted the lab work. JH Heng and ZH Wu conducted the statistical analysis. All authors read and approved the final manuscript.

## Supporting information


**Figure S1**

**Table S1**

**Supplementary Material**
