# Supplementary figures and images for: L‐carnitine increases cell proliferation and amino acid transporter expression via the activation of insulin‐like growth factor I signaling pathway in rat trophoblast cells
Source: Food Sci Nutr. 2020 Apr 28;8(7):3298–307. doi: 10.1002/fsn3.1607 (PMC7382193; doi:10.1002/fsn3.1607)

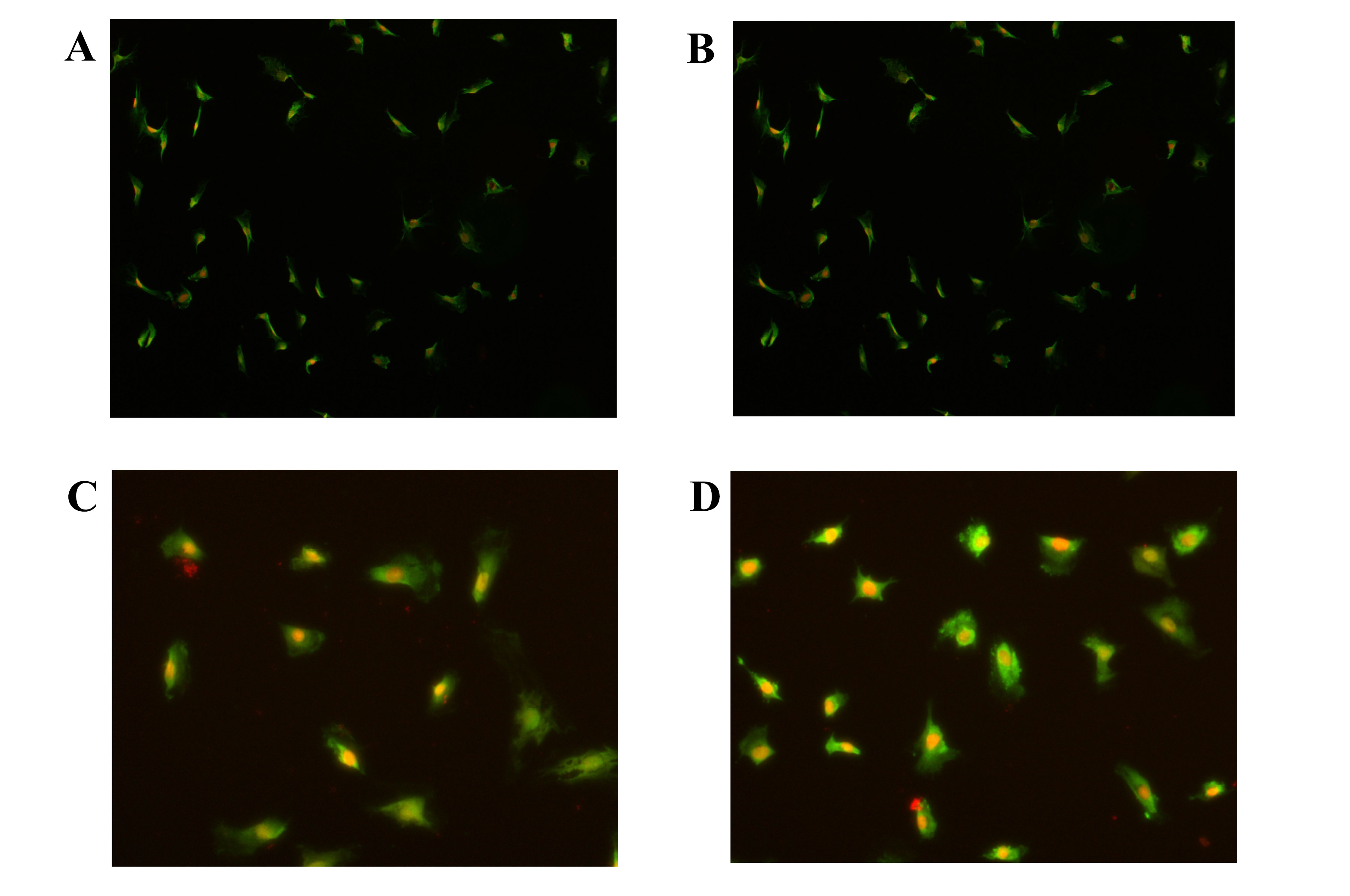

Supplement: Supplementary file 1 — Figure S1 [file FSN3-8-3298-s001.jpg]
