# Supplementary material for: L‐carnitine increases cell proliferation and amino acid transporter expression via the activation of insulin‐like growth factor I signaling pathway in rat trophoblast cells
Source: Food Sci Nutr. 2020 Apr 28;8(7):3298–307. doi: 10.1002/fsn3.1607 (PMC7382193; doi:10.1002/fsn3.1607)
Supplement: Supplementary file 2 — Table S1 [file FSN3-8-3298-s002.docx]

**Supplemental table 1.** Real-Time PCR Primer Sequence

| Primers | direction | Sequence (5’-3’) | Size |
| --- | --- | --- | --- |
| IGF-1 | forward | AGCGATGGGGAAAATCAGC | 197 bp |
|  | reverse | CATCCACAATGCCCGTCTG |  |
| IGF-1R | forward | CCGGCCTTTCACTCTGTACC | 161 bp |
|  | reverse | AGTTTTCAGGTCTTGGCTCCC |  |
| GLUT1 | forward | CCGCTTCCTGCTCATCAATC | 157 bp |
|  | reverse | CTGCCGACCCTCTTCTTTCA |  |
| GLUT3 | forward | GTGGCTCAGGTCTTTGGTTTG | 174 bp |
|  | reverse | CGCTTGGTCTTCCTCCTTTCT |  |
| CAT1 | forward | TGGCAGCTCACGGAGATTAA | 181 bp |
|  | reverse | AGCCCACGAAGGCATAAAAG |  |
| SNAT1 | forward | CCAGAGCACAGGCGACATTC | 113 bp |
|  | reverse | CCAGCTCAAACAACGAGGAC |  |
| SNAT2 | forward | CCTCCGGATCGATTACAATG | 147 bp |
|  | reverse | GGACCAGATAGTCGCCGTTC |  |
| SNAT4 | forward | TCCCGGAGAAAGATGCAGAC | 122 bp |
|  | reverse | TGGAGCAACTCGTCCTCAAC |  |
| GAPDH | forward | ATGGGAAGCTGGTCATCAAC | 221b p |
|  | reverse | CAGCGAGGCAAGGCTAGA |  |
