# Supplementary material for: L‐carnitine increases cell proliferation and amino acid transporter expression via the activation of insulin‐like growth factor I signaling pathway in rat trophoblast cells
Source: Food Sci Nutr. 2020 Apr 28;8(7):3298–307. doi: 10.1002/fsn3.1607 (PMC7382193; doi:10.1002/fsn3.1607)
Supplement: Supplementary file 3 — Supplementary Material [file FSN3-8-3298-s003.docx]

**Supplemental Figure 1** Immunofluorescent staining of rat placenta trophoblast cells. Fluorescence microscopy analysis of rat placenta trophoblast cells revealed that the isolated cells were strongly positive (green) for antibodies specific for vimentin (A: 100 ×; C. 200 ×) and cytokeratin(red) (B: 100 ×; D. 200 ×).
